# The association between Compulsory Community Treatment Order status and mortality in New Zealand

**DOI:** 10.1192/bjo.2022.629

**Published:** 2023-01-13

**Authors:** Ben Beaglehole, Giles Newton-Howes, Richard Porter, Chris Frampton

**Affiliations:** Department of Psychological Medicine, University of Otago, Christchurch, New Zealand; Department of Psychological Medicine, University of Otago, Wellington, New Zealand

**Keywords:** Mortality, compulsory treatment, diagnosis, coercion, antipsychotics

## Abstract

**Background:**

Compulsory Community Treatment Orders (CTOs) enable psychiatric medication without the need for consent. Careful scrutiny of outcomes including mortality is required to ensure compulsory treatment is evidence-based and ethical.

**Aims:**

To report mortality for patients placed on CTOs and analyse data according to CTO status, mortality cause and diagnosis.

**Method:**

Data for all patients placed under CTOs between 1 January 2009 and 31 December 2018 was provided by the Ministry of Health, New Zealand. Data included diagnostic and demographic information, dates of CTOs, and any dates and causes of death. Deaths were categorised into suicides, accidents and assaults, and medical causes. Mortality data are reported according to CTO status and diagnosis.

**Results:**

A total of 14 726 patients were placed on CTOs over the study period, during which there were 1328 deaths. The mortality rate was 2.97 on and 2.31 off CTOs (rate ratio 1.29, 95% CI 1.14–1.45; *P* < 0.01). The mortality rate for accidents and assaults was 0.44 on and 0.25 off CTOs (rate ratio 1.73, 95% CI 1.23–2.42; *P* < 0.01). The mortality rate for medical causes was 2.33 on and 1.90 off CTOs (rate ratio 1.22, 95% CI 1.07–1.40; *P* < 0.01). The suicide rate was 0.20 on and 0.15 off of CTOs (rate ratio 1.33, 95% CI 0.81–2.12; *P* = 0.22).

**Conclusions:**

Increased care and medication provided during compulsory treatment does not the modify the course of illness sufficiently to reduce mortality during CTOs. Higher mortality rates during CTO periods compared with non-CTO periods may reflect greater unwellness during CTOs.

Our goal is to report the relationships between Compulsory Community Treatment Order (CTO) status and key outcomes for individuals placed on CTOs by using whole of nation New Zealand databases.^1,2^ In previous analyses of all patients placed on CTOs over a 10-year period (*N* = 14 726), we reported that patients on CTOs receive more psychiatric community care and psychiatric medication (particularly depot antipsychotic medication) when on CTOs compared with non-CTO periods.^1^ We also reported distinct outcome signatures according to diagnosis. Reduced psychiatric admissions and admission days per annum were present on CTOs for patients with psychotic disorder diagnoses, whereas the opposite was found for non-psychotic disorder diagnoses (despite significantly greater psychiatric community care contacts and medication during CTOs for all).^2^ This study extends previous analyses by analysing new data on mortality for patients placed on CTOs over the same 10-year period. In New Zealand, patients placed on CTOs are required to accept treatment without the need for consent.^3^ Individual choice relating to medications and the receipt of care are often overridden. Involuntary treatments commonly include antipsychotic medications that are known to increase morbidity and mortality.^4–6^ Consequently, careful scrutiny of treatment, including examination of adverse outcomes such as mortality, is required to ensure treatment is effective and at least partially justify the ethical dilemma of potentially overriding refusal of consent.

## Previous studies

RCTs are not well-suited to evaluate rare outcomes such as mortality, typically because of limited study size populations and study duration. Cohort studies that include case registries provide an alternative means of evaluating CTO effectiveness.^7^ In these studies, patients on CTOs are often matched with a different group of patients receiving psychiatric care on a voluntary basis. This design allows the influence of time, regression to the mean and the natural history of psychiatric illness to be considered. However, the use of matched controls is limited by inherent difficulty in controlling for factors such as insight and refusal to accept treatment (factors that are not usually measured or accounted for in matched-control studies).^8^ This suggests that a within-persons design comparing outcomes for CTO recipients on and off CTO periods may be a more useful comparison. The relationship between community compulsory treatment and mortality has previously been considered. Kisely et al reported that patients who commenced CTOs had nearly half the rate of all-cause death compared with controls at 2 years after CTO initiation, with the greatest effects seen for deaths from cancer, cardiovascular disease and diseases of the central nervous system.^9^ Segal et al reported that CTOs commenced between 1990 and 2000 in the state of Victoria, Australia, were associated with reduced risk of death compared with a group that had not received compulsory community treatment.^10^ A repeat analysis for patients who commenced CTOs between 2000 and 2012 also reported reduced risk of death compared with a non-CTO cohort.^11^ In that study, the overall risk of death was reduced by 9% in the CTO cohort, although this comprised a 26% elevation in risk of death by external causes, assault or self-harm, and a 17% reduction in risk of death by other causes.^11^ In England, Hunt et al reported a lower suicide rate for patients who were subject to a community treatment order at the time of death compared with other patients.^12^ Barkhuizen et al also reported that mortality was lower for a cohort placed on CTOs (compared with a group not placed on CTOs) after a compulsory admission.^13^ This finding occurred alongside higher use of long-acting injectable antipsychotics. Each of the studies described above made comparisons between patients placed on CTOs with groups not receiving compulsory community treatment. The challenge in interpreting these findings is the inability of these studies to control for intangible factors, such as fluctuating levels of insight into illness and refusal to accept treatment. However, the reductions in mortality were reassuring, given the likelihood that increased severity of illness is present for patients treated under compulsion.

## The current study

Our research complements the approach taken by cohort studies described above by using a within-persons design. All patients receiving CTOs in New Zealand were categorised according to CTO status during the 10-year study period. Patients could be on and off CTOs on multiple occasions during the study period. We report whether outcomes are more or less frequent on CTOs compared with non-CTO periods for the group placed on CTOs over this 10-year period. It is reasonable to assume that CTOs are more likely to be applied during periods in which community mental illness is more severe. Therefore, our previous finding that admissions for patients with psychotic disorders reduced when on CTOs is a qualified marker of success for CTOs for patients in this diagnostic group. This study extends our previous research and further examines key outcomes associated with CTOs. Here, we report the association between CTO status and mortality for all patients receiving CTOs over a 10-year period in New Zealand.

## Method

The authors assert that all procedures contributing to this work comply with the ethical standards of the relevant national and institutional committees on human experimentation and with the Helsinki Declaration of 1975, as revised in 2008. All procedures involving human participants were approved by the Human Research Ethics Committee of the University of Otago (reference number HD19/076). This study analysed large databases. The data were received in anonymised form, using unique identifiers. As a consequence, informed consent was not required.

This study includes data from the previously reported data-set accessed from the Programme for the Integration of Mental Health Data (PRIMHD) database, containing all patients who commenced a CTO in New Zealand between 1 January 2009 and 31 December 2018.^1^ The PRIMHD data-set contains diagnostic and demographic information (age at first CTO commencement, gender, ethnicity, New Zealand Deprivation Index score), and dates of commencement and cessation of CTOs during the study period.^1^

The PRIMHD database provided the DSM-IV primary diagnoses for patients placed under CTOs during the 10-year study period. Patients could receive multiple diagnoses because of the potential for repeated contact with specialist mental health services over the study period. For this reason, organising principles were applied to categorise the diagnostic data. We did this using a hierarchical approach to create the following diagnostic groupings: dementia disorders, psychotic disorders, bipolar disorder type 1, other bipolar disorders, major depressive disorder, personality disorders, other diagnosis and no diagnosis. Further details of how this occurred are reported in our previous paper.^2^

For this study, we requested new data from the New Zealand Ministry of Health for individuals identified in the PRIMHD data-set and recorded in the Mortality Collection between 1 January 2009 and 31 December 2018. The underlying cause of death was provided by the Registrar of Births, Deaths and Marriages to the Ministry of Health, and recorded with ICD-10-AM (Australian Modification) codes in the Mortality Collection. Data was provided in anonymised form, using the same encrypted unique identifiers provided in the original PRIMHD data-set.

Mortality data was categorised according to total deaths and subgroups according to cause of death: suicide (codes X60–X84), accidents and assaults (codes V02–V04, V09.0–V09.3, V12–V14, V19.0–V19.2, V19.4–V19.6, V20–V79, V80.3–V80.5, V81.0–V81.1, V82.0–V82.1, V83.0–V83.3, V84.0–V84.3, V85.0–V85.3, V86.0–V87.8, V88.0–V88.8, V89.0, V89.2, V89.9, X85-Y09), and other medical causes (all other codes).

### Statistical analysis

Descriptive statistics were used to describe the study population. Mortality rates per 100 000-person years (subcategorised to suicide, accidents and assaults, and medical causes) were calculated for the periods on and off CTOs for the study population and for each diagnostic group. The grouped period for patients on CTOs was compared with the grouped period for patients off CTOs. This involved calculating the person-years represented for each individual on and off CTOs within the total study period. The total number of deaths both on and off CTOs for each individual were then summed to calculate the rates on and off of CTOs. A rate ratio was calculated with these incidences by dividing the mortality incidence on CTOs with the corresponding figure off CTOs. Rate ratios of <1 mean that mortality is less likely on CTOs and rate ratios >1 mean that mortality is more likely on CTOs. The 95% confidence intervals for the incidence and ratio estimates were calculated with standard Poisson approximation.^14^ Significant *P*-values were set at <0.05. SPSS version 27 for Windows was used for analysis.

## Results

A total of 14 726 patients were placed on a CTO over the 10-year period between 1 January 2009 and 31 December 2018. During this period, there were 1328 deaths. Three hundred and ninety-four deaths (29.6% of all deaths) during CTOs and 934 deaths during non-CTO periods. Approximately 25% of the total study period was spent on CTOs.

Table 1 describes the study population according to mortality grouping. The mean ages for the deceased population groups were older than the not-deceased group (mean age not-deceased 33.6 years, s.d. 14.8). The mean age for the death owing to medical causes group (mean age 55.2 years, s.d. 16.7) was older than the other groups. The death owing to accidents and assaults group were more likely to be male (67.5% male) than the other groups. Patients who died from suicide were more likely to be of New Zealand European ethnicity (67.4% New Zealand European ethnicity) than the other groups, and less likely to be Māori (20.2% Māori ethnicity). Mean New Zealand Deprivation Index scores ranged from 6.6 to 7.5, with higher deprivation observed in the not-deceased group (mean deprivation score 7.0, s.d. 2.6) and death owing to accidents and assaults group (mean deprivation score 7.5, s.d. 2.4). The lowest percentage of psychotic disorders was observed in the death owing to medical causes group (44.1%). The deceased groups were likely to be placed on more CTOs per annum than the not-deceased group (median CTOs per annum for deceased 0.3, interquartile range 0.2–0.6; median CTOs per annum for not-deceased 0.2, interquartile range 0.1–0.4).

**Table 1:** Description of the study population according to mortality status

Category	Not deceased (*n* = 13 398)	Deceased: all causes (*n* = 1328)	Deceased: accidents and assaults (*n* = 160)	Deceased: medical causes (*n* = 1079)	Deceased: suicide (*n* = 89)
Mean age, years (s.d.)	33.6 (14.8)	51.8 (18.0)	38.6 (17.2)	55.2 (16.7)	34.9 (12.2)
% Male	59.8	58.4	67.5	56.8	61.8
% New Zealand European	48.4	61.7	52.8	61.9	67.4
% Māori	34.3	28.4	34.8	27.8	20.2
Mean New Zealand Deprivation Index score (s.d.)	7.0 (2.6)	6.9 (2.5)	7.5 (2.4)	6.9 (2.5)	6.6 (2.5)
% Psychotic disorder	57.7	46.0	56.6	44.1	51.7
Median number of CTOs per annum (IQR)	0.2 (0.1–0.4)	0.3 (0.2–0.6)	0.4 (0.2–0.7)	0.3 (0.2–0.6)	0.4 (0.2–0.7)

CTO, Compulsory Community Treatment Order; IQR, interquartile range.

Table 2 reports overall mortality according to CTO status and diagnosis. The mortality rate for the study population was 2.97 on CTOs compared with 2.31 off CTOs (rate ratio 1.29, 95% CI 1.14–1.45; *P* < 0.01). The mortality rate for patients with psychotic disorders was 2.56 on CTOs and 1.77 off CTOs (rate ratio 1.45, 95% CI 1.22–1.70; *P* < 0.01). The mortality rate for the no diagnosis group was 4.24 on CTOs and 3.03 off CTOs (rate ratio 1.40, 95% CI 1.22–1.75; *P* < 0.01). There were no other significant findings according to diagnosis for overall mortality.

**Table 2:** Mortality for the CTO cohort, according to diagnosis and CTO status

Diagnostic group	Total deaths on CTOs	Total deaths off CTOs	Rate on CTOs	Rate off CTOs	Ratio (on/off)	95% CI lower limit	95% CI upper limit
Dementia disorders (*n* = 350)	7	81	3.73	7.43	0.50	0.20	1.08
Psychotic disorders (*n* = 8338)	230	381	2.56	1.77	1.45	1.22	1.71
Bipolar disorder type 1 (*n* = 1393)	27	87	3.11	2.06	1.51	0.94	2.34
Other bipolar disorders (*n* = 212)	5	25	4.43	3.78	1.17	0.35	3.12
Major depressive disorder (*n* = 417)	5	32	4.02	2.29	1.76	0.53	4.54
Personality disorders (*n* = 106)	0	6	0.00	1.77	0.00	−	−
Other diagnosis (*n* = 757)	10	52	2.73	2.17	1.26	0.57	2.50
No diagnosis (*n* = 3153)	110	270	4.24	3.03	1.40	1.11	1.75
Total (*N* = 14 726)	394	934	2.97	2.31	1.29	1.14	1.45

CTO, Compulsory Community Treatment Order.

Table 3 reports 160 deaths by accidents and assaults according to CTO status and diagnosis. The accidents and assaults mortality rate for the study population was 0.44 on CTOs and 0.25 off CTOs (rate ratio 1.73, 95% CI 1.23–2.42; *P* < 0.01). The accidents and assaults mortality rate for the no diagnosis group was 0.73 on CTOs and 0.20 off CTOs (rate ratio 3.63, 95% CI 1.80–7.33; *P* < 0.01). There were no other significant findings according to diagnosis for mortality by accidents and assaults.

**Table 3:** Mortality owing to accidents and assaults, according to diagnosis and CTO status

Diagnostic group	Deaths owing to accidents and assaults on CTOs	Deaths owing to accidents and assaults off CTOs	Rate on CTOs	Rate off CTOs	Ratio (on/off)	95% CI lower limit	95% CI upper limit
Dementia disorders (*n* = 350)	0	1	0.00	0.09	–	–	–
Psychotic disorders (*n* = 8338)	33	57	0.37	0.27	1.39	0.87	2.16
Bipolar disorder type 1 (*n* = 1393)	4	10	0.46	0.24	1.94	0.45	6.73
Other bipolar disorders (*n* = 212)	0	2	0.00	0.30	0.000	–	–
Major depressive disorder (*n* = 417)	0	3	0.00	0.21	0.000	–	–
Personality disorders (*n* = 106)	0	0	–	–	–	–	–
Other diagnosis (*n* = 757)	2	11	0.55	0.46	1.19	0.13	5.45
No diagnosis (*n* = 3153)	19	18	0.73	0.20	3.626	1.80	7.33
Total (*N* = 14 726)	58	102	0.44	0.25	1.734	1.23	2.42

CTO, Compulsory Community Treatment Order.

Table 4 reports 1079 deaths owing to medical causes during the 10-year study period. The medical causes mortality rate for all diagnoses was 2.33 on CTOs and 1.90 off CTOs (rate ratio 1.22, 95% CI 1.07–1.40; *P* < 0.01). The medical causes mortality rate for patients with psychotic disorders was 2.01 on CTOs and 1.37 off CTOs (rate ratio 1.46, 95% CI 1.21–1.76; *P* < 0.01). There were no other significant findings according to diagnosis for mortality by medical causes.

**Table 4:** Mortality owing to death by medical causes, according to diagnosis and CTO status

Diagnostic group	Deaths owing to medical causes on CTOs	Deaths owing to medical causes off CTOs	Rate on CTOs	Rate off CTOs	Ratio (on/off)	95% CI lower limit	95% CI upper limit
Dementia disorders (*n* = 350)	7	80	3.73	7.34	0.51	0.20	1.10
Psychotic disorders (*n* = 8338)	180	295	2.01	1.37	1.46	1.21	1.76
Bipolar disorder type 1 (*n* = 1393)	19	74	2.19	1.75	1.25	0.71	2.09
Other bipolar disorders (*n* = 212)	5	21	4.43	3.18	1.40	0.41	3.80
Major depressive disorder (*n* = 417)	2	20	1.61	1.43	1.12	0.13	4.62
Personality disorders (*n* = 106)	0	4	0.00	1.18	0.00	–	–
Other diagnosis (*n* = 757)	8	39	2.18	1.63	1.34	0.54	2.91
No diagnosis (*n* = 3153)	88	237	3.39	2.66	1.28	0.99	1.64
Total (*N* = 14 726)	309	770	2.33	1.90	1.22	1.07	1.40

CTO, Compulsory Community Treatment Order.

Table 5 reports 89 suicides during the study period. The suicide rate for the study population was 0.20 on CTOs and 0.15 off CTOs (rate ratio 1.33, 95% CI 0.81–2.12; *P* = 0.22). Significant findings for suicide by diagnosis were present for bipolar disorder type 1 and depressive disorders, but the absolute numbers of suicides for these diagnoses were small.

**Table 5:** Mortality owing to suicide, according to diagnosis and CTO status

Diagnostic group	Deaths from suicide on CTOs	Deaths from suicide off CTOs	Rate on CTOs	Rate off CTOs	Ratio (on/off)	95% CI lower limit	95% CI upper limit
Dementia disorders (*n* = 350)	0	0	–	–	–	–	–
Psychotic disorders (*n* = 8338)	17	29	0.19	0.14	1.40	0.72	2.64
Bipolar disorder type 1 (*n* = 1393)	4	3	0.46	0.07	6.47	1.10	44.20
Other bipolar disorders (*n* = 212)	0	2	0.00	0.30	0.000	–	–
Major depressive disorder (*n* = 417)	3	9	2.41	0.64	3.74	0.65	15.00
Personality disorders (*n* = 106)	0	2	0.00	0.59	0.00	–	–
Other diagnosis (*n* = 757)	0	2	0.00	0.08	0.00	–	–
No diagnosis (*n* = 3153)	3	15	0.12	0.17	0.69	0.13	2.43
Total (*N* = 14 726)	27	62	0.20	0.15	1.33	0.81	2.12

CTO, Compulsory Community Treatment Order.

## Discussion

We believe that there is an obligation to scrutinise key outcomes relating to CTOs because of the compulsory nature of care provided. If compulsory treatment is enforced, we believe that there is an expectation that better outcomes should result compared with voluntary treatment for CTO recipients. This study focuses on mortality according to CTO status for all New Zealanders placed on CTOs over a 10-year period. Overall mortality rates, deaths from accidents and assaults, and deaths from medical causes were all higher on CTOs compared with non-CTO periods. Findings relating to suicide were non-significant or with insufficient numbers to readily draw firm conclusions.

Our study compares grouped periods on CTOs with grouped periods off CTOs (the study cohort spent approximately 25% of the study period on CTOs). This design examines the association between CTOs and outcomes when they are *in situ* as opposed to other study designs that measure outcomes at a time point distant from CTO initiation (when CTOs may no longer be in place). It is likely that CTOs are enacted during periods of greater morbidity. As a consequence, higher mortality rates may simply reflect the fluctuating nature of illness and coincide with severity. However, we previously reported that CTOs were associated with less frequent psychiatric admissions and fewer psychiatric admission days per annum for patients with psychotic disorders (with the opposite occurring for non-psychotic disorder diagnoses).^2^ These findings occurred in association with higher rates of community care contacts and dispensing of psychiatric medication during CTOs for all diagnostic groups. It appeared that compulsory treatment provided through CTOs was effective in reducing admissions and enabling CTO recipients with psychotic disorders to remain in the community. When mortality is considered, however, there does not appear to be a similar beneficial association between compulsory treatment and outcome; deaths by accidents and assaults, and deaths from medical causes, were all higher during CTO periods across diagnoses.

Studies that make comparisons with control groups not placed on CTOs report that the initiation of a CTO is followed by lower mortality rates over the subsequent study period.^9–13^ These findings complement our own, but should be appraised in the context of the comparators. In our study, mortality rates were higher during CTOs, and comparison was made with the same group during non-CTO periods. For the other studies, CTOs were largely initiated at the time of an in-patient admission, and comparisons were made with a group not placed on CTOs (sometimes with matching of sociodemographic variables). Despite matching, it is likely that these groups differ significantly, at least in areas such as insight and willingness to accept treatment at the time discharge from hospital is planned. We suspect that between group differences explain some of the divergent findings between our study and the other studies reporting mortality associated with CTOs. Nonetheless, it is feasible that CTO recipients have lower rates of mortality compared with matched non-CTO recipients, and higher rates of mortality during CTOs compared with non-CTO periods.

Patients with serious mental illness are more likely to die by accidents and assaults than the general population.^15,16^ Our finding of higher mortality rates through accidents and assaults during CTOs adds to this literature. We suspect higher mortality through accidents and assaults when on CTOs occurs as a consequence of greater illness morbidity and increased vulnerability, despite increased community care during these times. Whether this would have been even higher had the patients not been compulsorily treated cannot be answered by this study design.

We previously reported that antipsychotic dispensing, including depot antipsychotic dispensing, occurs at much higher rates during CTOs.^1^ The finding was present for all diagnostic groups, despite the limited evidence base for depot antipsychotic medications in non-psychotic disorders.^2^ This study reports that rates of death by medical causes is higher when on CTOs. When death by medical causes is examined according to diagnosis, higher rates were present for each diagnostic group, with the exception of dementia disorders and personality disorders, but these findings were only significant for psychotic disorders. Given the association between antipsychotic medications, metabolic syndrome and sudden death owing to cardiac causes,^4–6^ higher rates of death by medical causes in our population are not unexpected. However, the possible contribution of compulsory antipsychotic treatment (without clear psychiatric indication in non-psychotic groups) to higher rates of death by medical causes will concern clinicians, patients and families. In New Zealand, CTOs are applied when an abnormal state of mind is present, characterised by psychiatric symptoms and serious risks to oneself or others or serious impairment in self-care.^3^ Although compulsory treatment is a necessary corollary of CTOs, these findings at least impose a burden on clinicians to focus on monitoring and medical treatment for those on CTOs.

The Office of the Chief Coroner, New Zealand, releases suicide data in New Zealand. For the period between 2009 and 2018 (corresponding with the study period), the suicide rate in New Zealand ranged between 10.8 and 12.4 per 100 000 population.^17^ Our study population had an annualised suicide rate of 60.43/100 000 population, which is a much higher risk than the general population. There were insufficient suicides between the diagnostic groups for conclusions to be drawn about the association between CTO status and diagnosis. However, as suicide risk is likely to be greater during CTO periods, the finding that suicides did not significantly increase for the study population during CTOs is reassuring.

The New Zealand Mental Health (Compulsory Assessment and Treatment) Act 1992 uses risk criteria as the basis for compulsory treatment.^3^ The finding of higher mortality rates during CTOs could therefore be interpreted as a justification for concerns leading to CTOs. Higher mortality rates could also be interpreted to mean that compulsory treatment provided during CTOs is ineffective in modifying at least some of the risk issues that led to CTO initiation. The ‘He Ara Oranga: Report of the Government Inquiry into Mental Health and Addiction’ examined the New Zealand Mental Health (Compulsory Assessment and Treatment) Act 1992 and found there is an urgent need to repeal and replace the act with a human rights-based act.^18^ Although compulsory treatment appears to reduce admissions for patients with psychotic disorders,^1,2^ we believe the current application of compulsory treatment to all diagnoses accompanied by higher mortality rates during CTOs is a signal that continued scrutiny on the form and extent of compulsory treatment is required.

### Limitations

Mortality is a definitive outcome measure suited to quantitative analysis. However, the use of routinely collected national data means that some information, such as illness severity, is unavailable. Our analysis compared the grouped period on CTOs with the grouped period off of CTOs for CTO recipients. This enables robust outcome rates for mortality and rate ratios to be calculated, but this approach was not amenable to standard multivariate analysis. Data describing the patients age, gender, ethnicity and diagnosis were available. However, other confounding factors likely to affect mortality, such as illness severity, illness history and physical and mental comorbidity, were not collected. Prospective, longitudinally designed studies are required to address this limitation and explore these effects on mortality. We also acknowledge that the complexity of CTO treatment requires a multifaceted approach, and that qualitative work in this area is required to better place our findings in context. The no diagnosis group comprised 21.3% of the study population. Diagnostic characteristics are not present in PRIMHD for this group, which we suspect relate to reporting limitations known to be present in the database^19^ as opposed to this group not being given diagnoses by treating clinicians.

In conclusion, for New Zealanders placed on CTOs over a 10-year period, higher mortality rates and more frequent deaths by accidents and assaults, and medical causes, are present during CTOs compared with non-CTO periods. These findings should be interpreted alongside our previous work reporting more frequent community care and greater use of antipsychotic medication during CTOs, and less frequent admissions for patients with psychotic disorders on CTOs. Higher mortality during CTOs is concerning, particularly when the juxtaposition of higher rates of psychiatric treatment and death from medical causes is considered. We therefore believe this study highlights the importance of attention, care and monitoring for this potentially vulnerable group of patients, as well as legislative reform that prioritises patient autonomy whenever possible.

## Data Availability

The corresponding author, B.B., will support access to the aggregated data-set from which this study is drawn, upon reasonable request.
